# Spatial distributions, feeding ecologies, and behavioral interactions of four rabbitfish species (*Siganus unimaculatus*, *S. virgatus*, *S. corallinus*, and *S. puellus*)

**DOI:** 10.7717/peerj.6145

**Published:** 2018-12-20

**Authors:** Atsushi Nanami

**Affiliations:** Research Center for Sub-tropical Fisheries, Seikai National Fisheries Research Institute, Japan Fisheries Research and Education Agency, Ishigaki, Okinawa, Japan

**Keywords:** Coral reef, Foraging substrates, Food item, Species coexistence, Body shape characteristics, Environmental characteristics, Spatial distribution, Rabbitfish, Conspecific aggression

## Abstract

Clarifying the underlying mechanisms that enable closely related species to coexist in a particular environment is a fundamental aspect of ecology. Coral reefs support a high diversity of marine organisms, among which rabbitfishes (family Siganidae) are a major component The present study aimed to reveal the mechanism that allows rabbitfishes to coexist on coral reefs in Okinawa, Japan, by investigating the spatial distributions, feeding ecologies, and behavioral interactions of four species: *Siganus unimaculatus, S. virgatus, S. corallinus*, and* S. puellus*. All four species had a size-specific spatial distribution, whereby small individuals were found in sheltered areas that were covered by branching and bottlebrush *Acropora* spp. and large individuals were found in both sheltered and exposed rocky areas. However, no clear species-specific spatial distribution was observed. There was some variation in the food items taken, with *S. unimaculatus* primarily feeding on brown foliose algae, red foliose algae, and red styloid algae, and *S. virgatus* and *S. puellus* preferring brown foliose algae and sponges, respectively. However, *S. corallinus* did not show any clear differences in food preferences from *S. virgatus* or *S. unimaculatus*, mainly feeding on brown foliose algae and red styloid algae. The four species exhibited differences in foraging substrate use, which was probably related to differences in their body shape characteristics: *S. unimaculatus* has a slender body with a remarkably protruding snout and mainly used concave substrates for feeding, whereas *S. virgatus* has a deeper body with a low degree of snout protrusion and mainly used convex substrates. The other two species have a low degree of snout protrusion combined with a deeper body in the case of *S. corallinus* and a slender body in the case of *S. puellus* and used concave, flat, and convex substrates to an equal degree for feeding. Behavioral interactions were categorized into “agonistic behaviors” (attack and agonistic displays) and “no interactions.” For all four species, a greater frequency of agonistic behaviors was observed when two conspecific pairs approached each other than when two heterospecific individuals encountered each other. Together, these results suggest that food item partitioning is one of the main factors enabling the coexistence of these four syntopic rabbitfish species, which is enhanced by species-specific differences in feeding substrates as a result of their different body shape and behavioral characteristics.

## Introduction

Clarifying the mechanisms that allow species to coexist in a particular environment is a fundamental aspect of ecology (Knowlton & Jackson, 2001). It is generally considered that this coexistence is achieved through resource partitioning, particularly of habitat types and food items, via inter- and intraspecific competition (Tokeshi, 1999).

Coral reefs support a high diversity of marine organisms and it has been shown that coral reef fishes exhibit species-specific habitat partitioning (Russ, 1984; Williams, 1991; Munday, Jones & Caley, 1997; Donaldson, 2002; Nanami et al., 2005; Jankowski, Gardiner & Jones, 2015; Eurich, McCormick & Jones, 2018) as well as size-specific habitat partitioning (Beets & Hixon, 1994; Green, 1996; Claisse et al., 2009; Nanami et al., 2013). Therefore, habitat characteristics (e.g., coral morphology and coverage, wave exposure, and water depth) appear to be major determinants of both the species-specific and size-specific spatial distributions of coral reef fishes.

Several studies have also demonstrated that species-specific food item and foraging substrate (i.e., feeding microhabitat) partitioning enable the coexistence of diverse fish species in the absence of distinct habitat partitioning (Gradfelter & Johnson, 1983; Bouchon-Navaro, 1986; Pratchett, 2005; Liedke et al., 2017). In some cases, the morphological characteristics of a species (e.g., body shape, dentition type, and jaw-lever mechanics) affect this partitioning among syntopic species (Carpenter, 1996; Nanami & Shimose, 2013; Brandl, William & Bellwood, 2015). Therefore, clarifying the relationship between food item partitioning and this so-called trophic morphology is critical to understanding the mechanism that underlies species coexistence.

It is also important to clarify the inter- and intraspecific interactions that occur among multiple species when competing for resources, as some studies have suggested that heterospecific and/or conspecific aggression enhance the effects of habitat partitioning (Bay, Jones & McCormick, 2001; Munday, Jones & Caley, 2001; Medeiros, Souza & Ilarri, 2010; Geange, Stier & Shima, 2013; Eurich, McCormick & Jones, 2018).

A high species richness of fishes is considered important for maintaining viable coral reef ecosystems (Brandl, Hoey & Bellwood, 2014; Brandl & Bellwood, 2016). The diverse fish species that are found in coral reefs include a number of herbivorous fishes, such as parrotfishes (family Scaridae), surgeonfishes (family Acanthuridae), and rabbitfishes (family Siganidae). Some surgeonfishes and rabbitfishes primarily target benthic macroalgae for feeding, whereas parrotfishes mainly feed on epilithic algae (Choat, Clements & Robbins, 2002; Hoey, Brandl & Bellwood, 2013; Clements et al., 2016). These herbivorous fishes are considered to play various roles in controlling coral–algal interactions on the hard substrates of coral reefs via their grazing, cropping, and browsing activities (Bellwood et al., 2004; Fox & Bellwood, 2008; Hoey & Bellwood, 2008; Brandl & Bellwood, 2013; Bonaldo, Hoey & Bellwood, 2014). Among these, rabbitfishes have recently attracted attention for not only their functional role but also their ability to coexist with closely related species.

Fox & Bellwood (2013) showed that rabbitfishes on the Great Barrier Reef had very different feeding microhabitats from other herbivorous species, such as parrotfishes and surgeonfishes. Furthermore, Hoey, Brandl & Bellwood (2013) showed that 11 rabbitfish species on this reef exhibited species-specific variations in diet composition and spatial distribution, and Fox et al. (2009) demonstrated that two closely related rabbitfishes had clear differences in diet composition and feeding periods. Thus, it appears that feeding niche partitioning is an important component of coexistence among herbivorous species. However, the ecology of rabbitfishes on coral reefs around Okinawa, Japan, is not well understood, despite these fishes being a major component of the fish assemblages here. Furthermore, the species-specific spatial distributions of coral reef rabbitfishes in relation to size and the relationship between feeding substrate and body shape characteristics remain to be clarified, as does the effect of behavioral interactions, particularly agonistic aggression, on the coexistence of these species.

The aim of the present study was to investigate the spatial distributions, feeding ecologies, and aggressive behavioral interactions among four species of rabbitfishes inhabiting an Okinawan coral reef. Specifically, it sought to clarify (1) the size-specific spatial distribution of the four rabbitfish species in relation to environment characteristics, (2) inter-specific differences in food items, (3) inter-specific differences in feeding substrates in relation to body shape characteristics, and (4) the level of inter- or intraspecific aggression.

## Materials and Methods

This study mainly involved observing free-living fishes in their natural habitat. Individuals that were caught by spearing for sampling were immediately killed by placing them on ice to minimize pain. The sampling procedure was approved by the Okinawa Prefectural Government in compliance with the fisheries coordination regulation no. 41, which permits the capture of marine fishes on Okinawan coral reefs for scientific purposes.

### Study species

**Figure 1:**
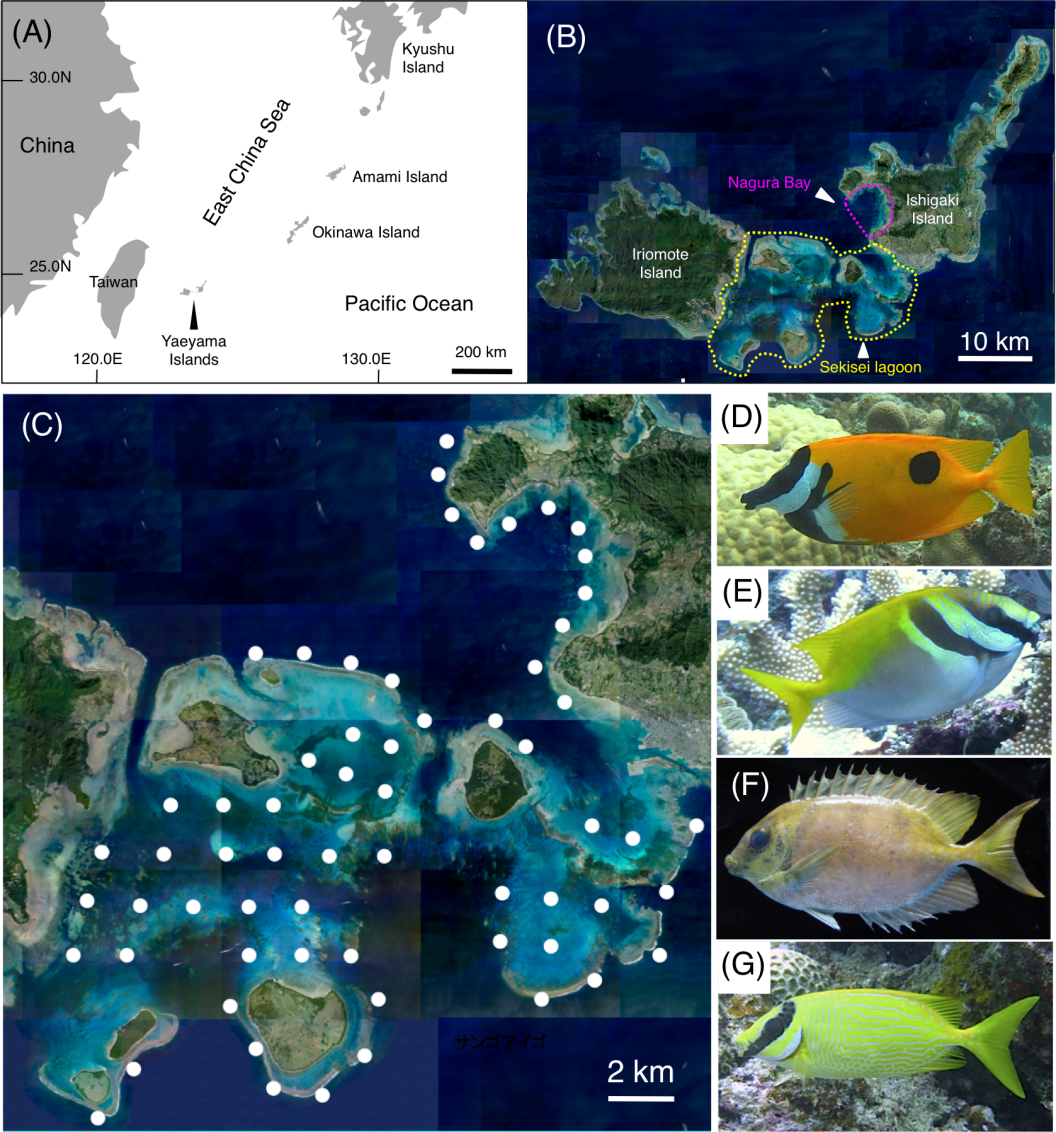
Study site and the four rabbitfish species. Maps showing the positions of the Yaeyama Islands (A), Sekisei Lagoon and Nagura Bay (B), and the 63 study sites used for underwater observation (C), and photographs of the four study species (D: *Siganus unimaculatus*; E: *S. virgatus*; F: *S. corallinus*; G: *S. puellus*). The aerial photographs used in (B) and (C) were provided by the International Coral Reef Research and Monitoring Center. The photographs of the four rabbitfish species (D–G) were taken by the author (A. Nanami).

Four rabbitfish species were examined in the present study (*Siganus unimaculatus, S. virgatus*, *S. corallinus*, and *S. puellus*; Figs. 1D–1G), all of which are commonly found in the Okinawan region (Masuda & Kobayashi, 1994).

### Spatial distributions of the fishes

To clarify the size-specific spatial distributions of the four rabbitfish species, underwater visual observations were conducted at Sekisei Lagoon and Nagura Bay in the Yaeyama Islands, Okinawa, in the southern part of the East China Sea (Figs. 1A, 1B). To obtain a more generalized understanding of their spatial distributions, observations were conducted during two periods: between June 2016 and January 2017, and between June 2017 and February 2018. Both series of observations were carried out at 63 study sites with an inter-site distance of ca. 2 km, allowing almost the entire area of Sekisei Lagoon and Nagura Bay to be surveyed (Fig. 1C). At each site, a 20-minute underwater visual survey was conducted along a 5-m-wide transect between 0830 and 1600 h using SCUBA equipment, following the methods of Nanami et al. (2013). During each 20-minute survey, a portable global positioning system receiver was attached to a buoy that was towed, allowing the distance covered to be recorded. At each site, the number of individuals observed and their total length (TL) were recorded for each of the four rabbitfish species. The number of individuals was then converted into a density (number of individuals per 100 m ×5 m) using the distance data. The average distances covered were 343.6 ± 43.8 m (mean ±  standard deviation (SD)) and 370.6 ± 54.7 m for the first and second series of observations, respectively.

A diving computer (Xtender Quattro; SCUBAPRO) was used to record the depth profile at 30-second intervals and PCLogBook software (SCUBAPRO) was then used to download the depth profile to a personal computer. This yielded 40 depth values for each site (two depth points per minute ×20 min), which were averaged for analysis. The water depths ranged from 2.6 m to 12.4 m and from 3.2 m to 12.1 m in the first and second series of observations, respectively.

### Spatial variation in substrate characteristics and depth at the study sites

To evaluate the substrate availability at each site, digital video images (moving pictures) of the substrate were recorded. Static images were then obtained at 10-second intervals using QuickTime Player Pro software (version 7.6), yielding 121 static images per 20-minute video image (from 0 to 1,200 s). The substrate at the center of each static image on the monitor of a personal computer was recorded for analysis. The substrate was divided into 16 categories for analysis, following Nanami et al. (2013) with some modification: (1) branching *Acropora* spp. (e.g., *A. formosa*), (2) tabular *Acropora* spp. (e.g., *A. hyacinthus*), (3) bottlebrush *Acropora* spp. (e.g., *A. carduus*), (4) branching corals except for *Acropora* spp. (e.g., branching *Pocillopora* spp., *Montipora* spp., and *Porites* spp.), (5) massive corals (e.g., massive *Porites* spp. and Faviidae), (6) other living corals (e.g., encrusting corals and leafy corals), (7) dead branching *Acropora* spp., (8) dead tabular *Acropora* spp., (9) dead bottlebrush *Acropora* spp., (10) dead branching corals except for *Acropora* spp., (11) dead other corals, (12) soft corals, (13) coral rubble, (14) rocks (calcium carbonate substratum with a lower substrate complexity), (15) sand, and (16) seaweed (e.g., *Padina minor* and *Sargassum* spp.). Both dead corals and rocks were covered by epilithic algae (Nanami, 2016).

### Spatial distribution analysis

Individual fish were categorized into three size classes: small (TL ≤ 10 cm), medium (11 cm ≤ TL ≤ 15 cm), and large (TL ≥ 16 cm). The relationship between the spatial distribution of each size class for the four rabbitfish species and environmental characteristics (16 substrates and depth) was analyzed using canonical correspondence analysis (CCA) with CANOCO software (Ter Braak & Smilauer, 2002). This analysis was performed using averaged data from the two series of observations. To extract the environmental characteristics that significantly affected the spatial distribution, software options set for forward selection were applied.

### Stomach contents analysis

To clarify the inter-specific differences in food items taken, the four rabbitfish species were sampled between February and August 2014 as follows: *Siganus unimaculatus*, *n* = 10, fork length (FL) = 149.0–166.0 mm, sampling period = February–July; *S. virgatus*, *n* = 10, FL = 159.5–215.5 mm, sampling period = June–July; *S. corallinus*, *n* = 10, FL = 126.0–205.5 mm, sampling period = April–August; and *S. puellus*, *n* = 10, FL = 148.0–219.0 mm, sampling period = April–August). Each individual was collected by spearing the head and was immediately placed in an icebox to minimize pain and any loss of stomach contents.

The stomach was dissected from each specimen and any food items in the stomach were recorded. The food items were sorted into 12 categories based on Hoey, Brandl & Bellwood (2013) with some modification: (1) brown foliose algae, (2) brown styloid algae (brown algae with a fine stalk-like shape), (3) brown filamentous algae, (4) other brown algae (brown algae that could not be sorted into the above-mentioned categories), (5) red foliose algae, (6) red styloid algae (red algae with a fine stalk-like shape), (7) red filamentous algae, (8) other red algae (red algae that could not be sorted into the above-mentioned categories), (9) green algae, (10) seagrass, (11) sponges, and (12) benthic invertebrates except for sponges (including Amphipoda, Anthozoa, Ascidiacea, Bivalvia, Bryozoa, Copepoda, Decapoda, Gastropoda, Hydrozoa, Isopoda, Ophiuroidea, Ostracoda, Polychaeta, and Radiolaria). Each food item was spread on a mesh sheet (mesh size = 5 mm ×5 mm) taking care not to overlay one item on another and the number of mesh intervals covered by each food item was counted. The number of mesh intervals covered was regarded as the volume of the food item.

The degree of similarity of food items among the 40 individuals (4 species ×10 individuals) was determined by calculating the Bray–Curtis similarity coefficient based on the percentage of each food item using PRIMER software (version 6). A dendrogram was then produced using the group-average linkage method.

### Feeding behavior analysis

To examine inter-specific differences in feeding substrates, the feeding behaviors of the four species were observed around Ishigaki Island in December 2013 and data on the feeding substrates were also recorded. The estimated TL (cm), number of bites per 5 min (feeding rate), and number of bites during a continuous feeding period (foraging bouts; sensu (Bellwood & Choat, 1990) were counted using SCUBA equipment or snorkeling between 0900 and 1600 h. The numbers of individuals and TL ranges were as follows: *S. unimaculatus*, *n* = 17, TL = 16.0–20.0 cm; *S. virgatus*, *n* = 16, TL = 16.0–22.0 cm; *S. corallinus*, *n* = 11, TL = 19.0–26.0 cm; and *S. puellus*, *n* = 17, TL = 15.0–23.0 cm.

Preliminary observations revealed that the feeding substrates were almost completely limited to dead corals and rocks. Therefore, they were categorized according to structural features (i.e., concave, flat, or convex) rather than microhabitat (e.g., branching corals, massive corals, or tabular corals). The feeding substrates were classified into three categories, following Bellwood & Choat (1990): (1) concave substrate (e.g., inter-branch space of dead branching coral colonies and dents in rocks), (2) flat substrate (e.g., surface of dead tabular coral colonies and flat-shaped rocks), and (3) convex substrate (e.g., surface of dead massive coral colonies).

The feeding rates and numbers of foraging bouts on each of the three substrates among the four species were compared using one-way analysis of variance (ANOVA) followed by the post-hoc Games-Howell test.

### Body shape analysis

To examine the relationship between feeding substrates and body shape characteristics, inter-specific body shape variations were analyzed using elliptic Fourier descriptors, which allow the shape of a closed two-dimensional contour to be analyzed (Iwata & Ukai, 2002) (see Furuta et al. (1995), Iwata et al. (1998), Iwata & Ukai (2002), and Nanami & Shimos (2013) for details). Briefly, the contour of the fish body shape was traced from an arbitrary point on the body surface around the body of the fish to return to the start point and was then projected on horizontal (*x*) and vertical (*y*) axes, allowing the contours along the *x*- and *y*-coordinates to be obtained. These contours were expressed as a waveform and approximated using the elliptic Fourier expansion. The body shape characteristics were summarized using principal component analysis (PCA). The estimated body shape was drawn against three values (−2 SD, the average, and +2 SD) of the principal component scores for PCA axes 1 and 2 using the obtained Fourier coefficients. This estimated body shape was then used to recognize the relationship between the two PCA axes and the body shape.

The analyses were conducted using SHAPE software version 1.3 (Iwata & Ukai, 2002). Ten individuals were analyzed for each species with the following FL ranges: *S. unimaculatus*, 147.0–188 mm; *S. puellus*, 148.0–195 mm; *S. corallinus*, 162.5–212.0 mm; and *S. virgatus*, 155.0–215.5 mm. The shape of the lateral aspect of each fish (excluding the dorsal, pelvic, anal, and caudal fins) was used in the analysis. To examine inter-specific differences in body shape characteristics, the PCA scores for PCA axes 1 and 2 were compared using one-way ANOVA followed by the post-hoc Games-Howell test for multiple comparisons among the four species.

### Behavioral interactions

To examine the behavioral characteristics of the four rabbitfish species during inter- and intraspecific encounters, aggressive interactions were recorded at the study site by following pairs of fish by snorkeling or using SCUBA equipment. First, an observer approached a focal pair (two individuals swimming within 50 cm of each other with no aggressive behavior) or a solitary individual, taking care not to scare them (the distance between the focal pair and observer was always >3 m). The fish were then directly observed for 5–10 min. Whenever the focal pair (or individual) encountered other pairs or individuals, the observer recorded the species that was encountered and any occurrence of (1) an attack (chasing other pairs or individuals), (2) an agonistic display (approaching other pairs or individuals and showing fin displays, such as raising the dorsal, pelvic, and anal fins), and (3) no interaction (no noticeable change in behavior when pairs were within 50 cm of each other), following Nanami (2015). At the end of the observation period, a new focal pair or individual was chosen taking care not to choose the same pair or individual.

The frequency of occurrence of the three types of behaviors was calculated for each pair. For the purposes of analysis, “attack” and “agonistic display” were combined and regarded as “agonistic behavior,” giving two categories of inter- and intraspecific interactions: (1) agonistic behavior and (2) no interaction. The ratios of the two behavioral categories were compared using one-way ANOVA followed by post-hoc Scheffé tests for multiple comparisons among the four species.

## Results

### Spatial distributions

**Figure 2:**
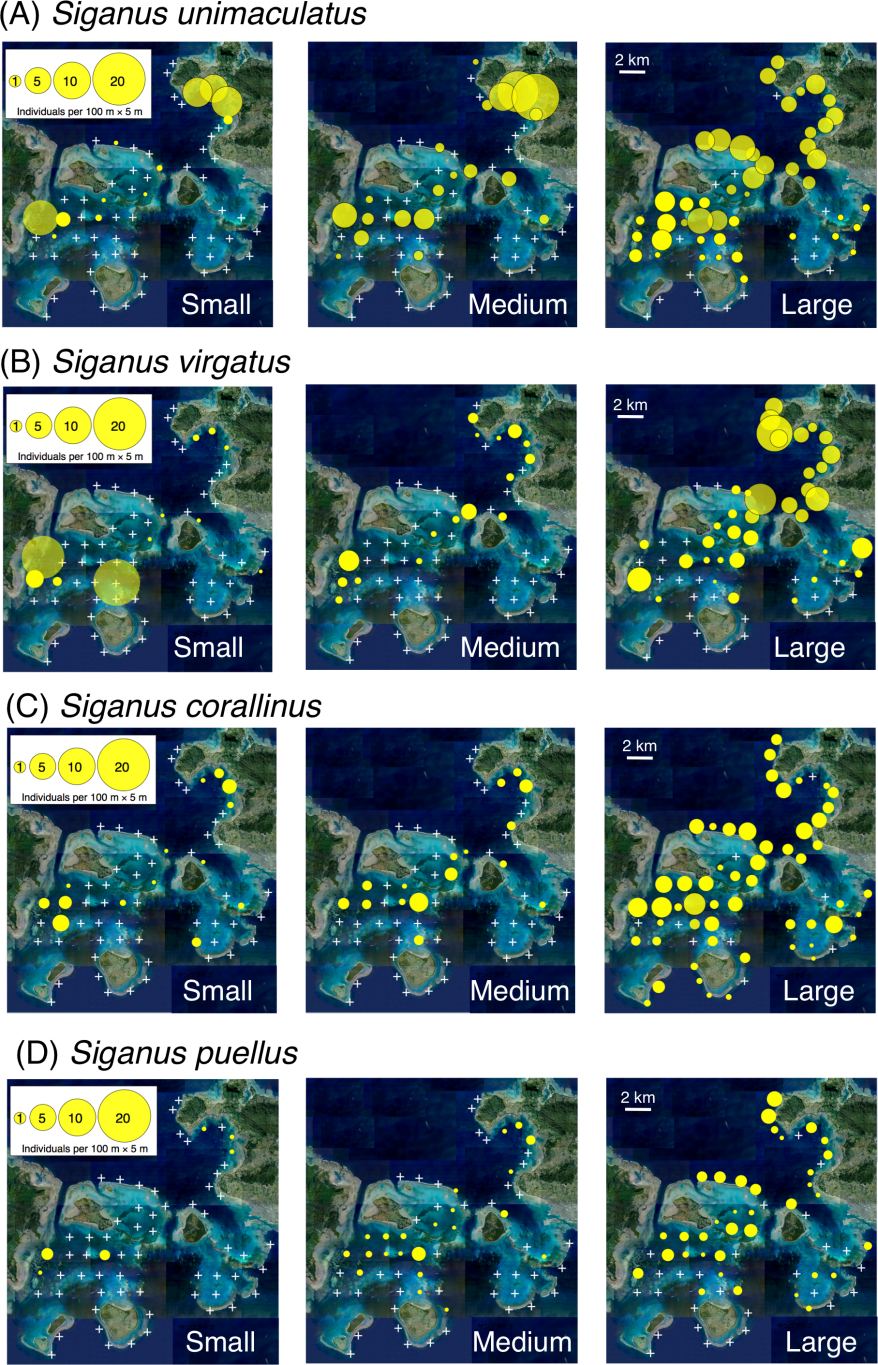
Spatial distributions of the four rabbitfish species. (A) *Siganus unimaculatus*, (B) *Siganus virgatus*, (C) *Siganus corallinus*, (D) *Siganus puellus*. Averaged data from the two series of observations were used in the analysis. Individuals of each species were categorized into three size classes: small, total length (TL) ≤ 10 cm; medium, 11 cm ≤ TL ≤ 15 cm; and large, TL ≥ 16 cm. White crosses represent no individuals. The aerial photograph was provided by the International Coral Reef Research and Monitoring Center.

Small individuals of all four species had a relatively limited distribution through the study area (e.g., the inner area of Nagura Bay and western area of Sekisei Lagoon), whereas large individuals had a more extensive distribution and medium-sized individuals had an intermediate distribution (Fig. 2).

**Figure 3:**
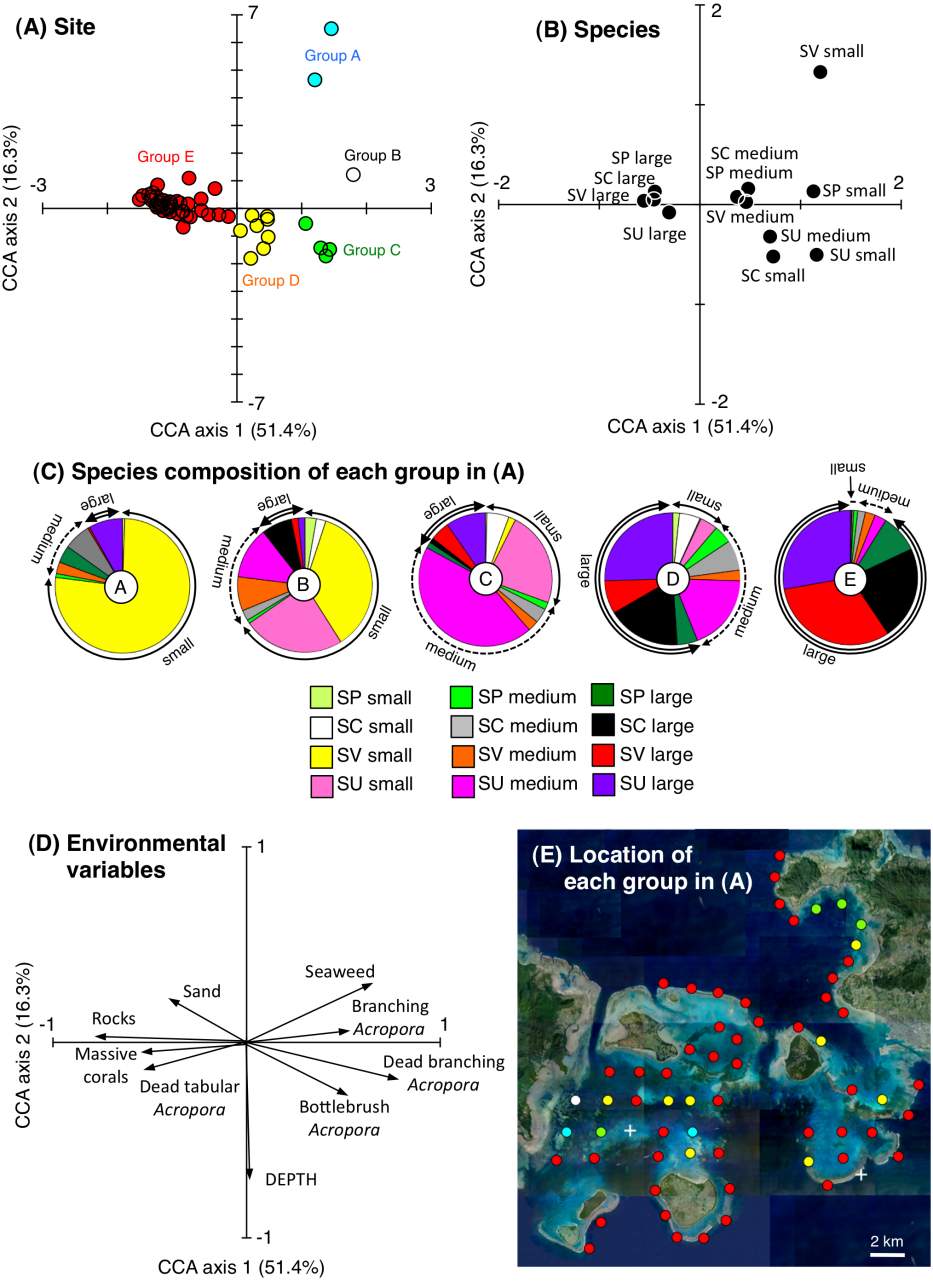
Results of the canonical correspondence analysis (CCA) to explain the relationship between the spatial distribution of the four rabbitfish species and environmental characteristics. Averaged data from the two series of observations were used in the analysis. The species compositions of the five groups in (A) are shown in (C). Only those environmental variables that had a significant effect on the spatial distributions of the fishes are shown in (D). Site locations for the five groups in (A) are plotted on an aerial photograph (as five different colored circles) in (E) to identify the location of each group. White crosses in (E) represent no individuals. The aerial photograph was provided by the International Coral Reef Research and Monitoring Center. In (B) and (C), species names are shown as abbreviations (Sc: *Siganus corallinus*, Su: *S. unimaculatus*; Sv: *S. virgatus*; Sp: *S. puellus*).

CCA revealed that the 63 study sites could be divided into five groups based on the species- and size-specific spatial distributions (Figs. 3A–3C): group A, which included two sites with a higher density of small *S. virgatus* individuals; group B, which included one site with a higher density of small *S. virgatus* and *S. unimaculatus* individuals; group C, which included four sites with a higher density of small, medium, and large *S. unimaculatus* individuals; Group D, which included eight sites with a higher density of medium and large individuals of all four species; and Group E, which included 48 sites with a higher density of large individuals of all four species.

Group A consisted of relatively shallow sites with a greater coverage of branching *Acropora* spp. and seaweed, Group B consisted of sites with a greater coverage of branching *Acropora* spp., Group C consisted of relatively deep sites with a greater coverage of bottlebrush *Acropora* spp. and dead *Acropora* spp., and group E consisted of sites with a greater coverage of rocks (Fig. 3D). Overall, group A, B and C sites were mainly located in sheltered parts of the study area (the inner areas of Nagura Bay and Sekisei Lagoon), whereas group E sites were located in both exposed (outer edge of the study area) and sheltered regions (Fig. 3E).

### Diets

**Figure 4:**
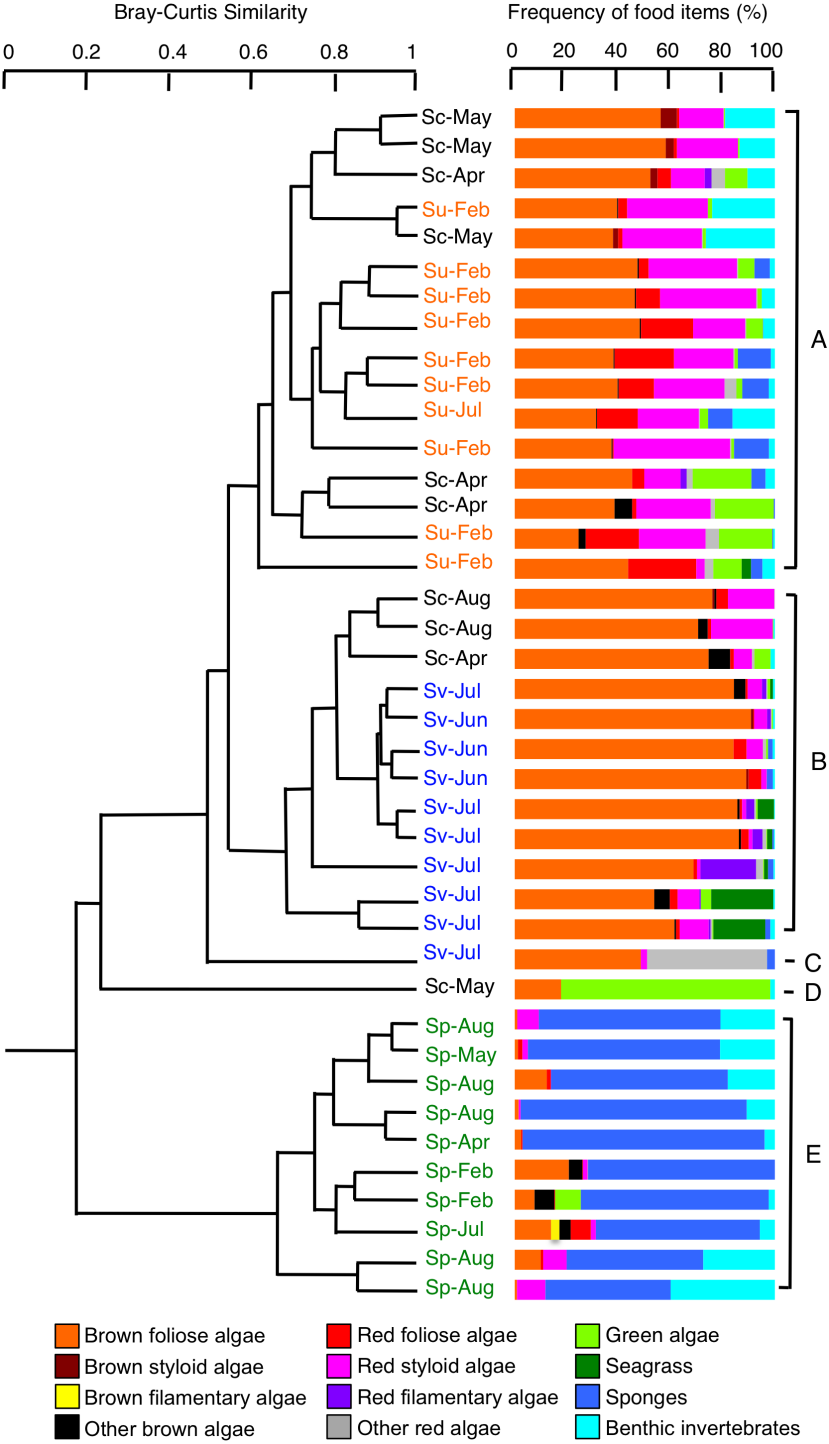
Dendrogram for the hierarchical clustering of the 40 rabbitfish individuals (4 species × 10 individuals) based on the similarity of food items (group-average linkage method using the Bray–Curtis similarity index). Species names are abbreviated as follows: Sc, *Siganus corallinus*; Su, *S. unimaculatus*; Sv, *S. virgatus*; Sp, *S. puellus*. Sampling months are shown alongside the species names. See Table S1 for details on the benthic invertebrates.

Cluster analysis revealed that all 10 individuals of *S. unimaculatus* mainly fed on brown foliose algae, red foliose algae, and red styloid algae, with sponges and bryozoa also being found to some extent (Fig. 4, Table S1). By contrast, the main food item of *S. virgatus* was brown foliose algae. The main food items of *S. corallinus* varied between individuals. Overall, brown foliose algae and red styloid algae were taken but hydrozoans and green algae were also found to some extent (Fig. 4). According to the cluster analysis, the 10 *S. corallinus* individuals were divided into three groups: a group in which *S. unimaculatus* predominated (six individuals; group A in Fig. 4), a group in which *S. virgatus* predominated (three individuals; group B), and the remainder (one individual; group D). All 10 individuals of *S. puellus* mainly had sponges in their stomachs (Fig. 4) but bryozoa and hydrozoa were also taken to some extent (Table S1), indicating that this species had a very different diet from the other three species.

### Feeding behaviors

**Figure 5:**
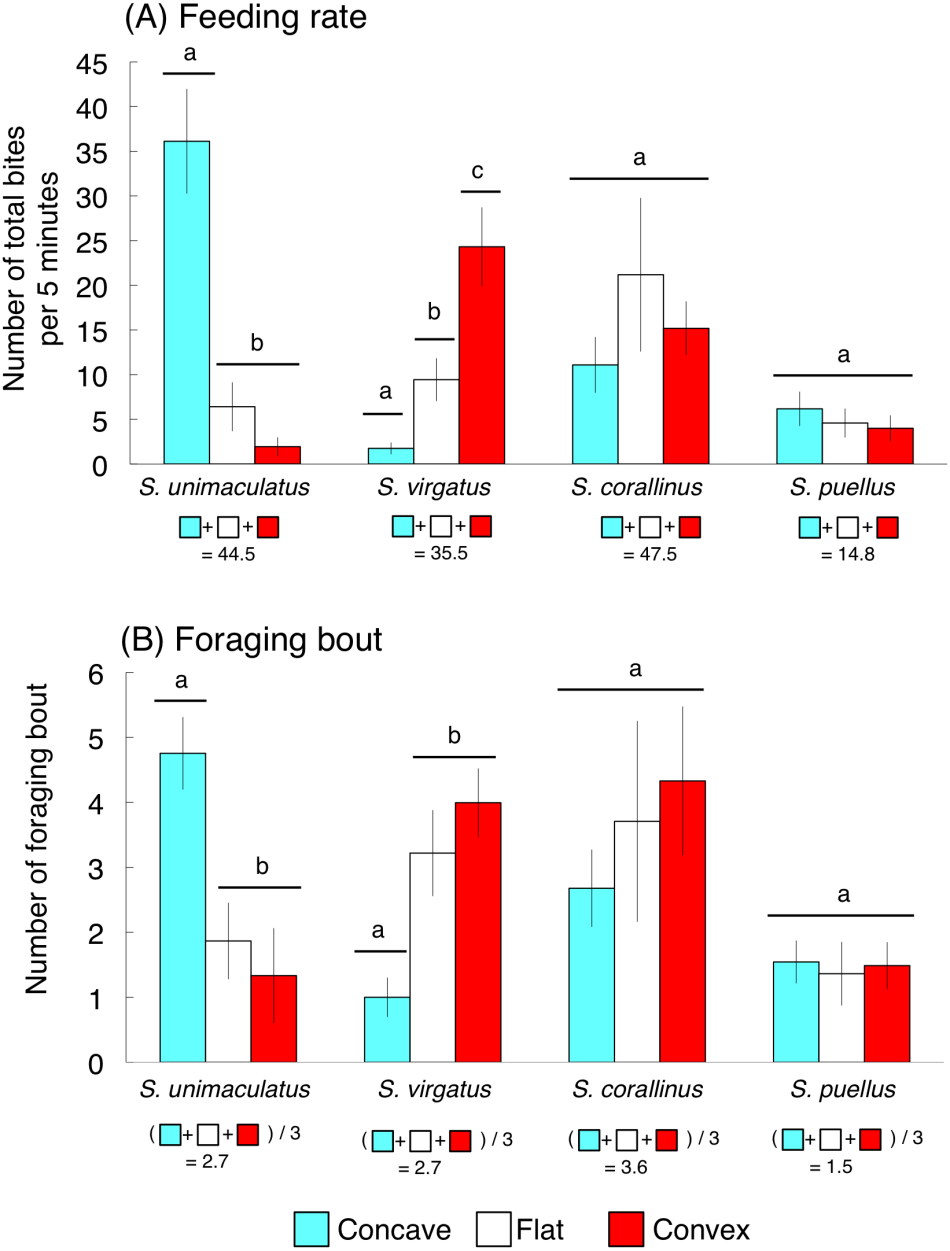
Feeding rates and numbers of foraging bouts (number of bites during a continuous feeding period) for the four rabbitfish species. (A) Feeding rate. (B) Foraging bout. For each species, bars that are connected by a line with the same lower-case letter are not significantly different (Games-Howell test, *p* > 0.05). Vertical bars represent standard deviations.

The average feeding rates for *S. unimaculatus, S. virgatus*, *S. corallinus*, and *S. puellus* were 44.5, 35.5, 47.5, and 14.8 bites per 5 min, respectively (Fig. 5A). *Siganus unimaculatus* mainly used concave substrates whereas *S. virgatus* mainly used convex substrates for feeding (one-way ANOVA and Games-Howell test, *p* < 0.05). However, no significant differences in feeding substrate were found for *S. corallinus* and *S. puellus* (Fig. 5A).

The average numbers of foraging bouts for *S. unimaculatus, S. virgatus*, *S. corallinus*, and *S. puellus* were 2.7, 2.7, 3.6, and 1.5 bites/bout, respectively (Fig. 5B). The number of foraging bouts was significantly higher on concave substrates for *S. unimaculatus* and convex and flat substrates for *S. virgatus* (one-way ANOVA and Games-Howell test, *p* < 0.05). Again, there were no significant differences in the number of foraging bouts among substrates for *S. corallinus* and *S. puellus* (Fig. 5B).

### Body shape characteristics

**Figure 6:**
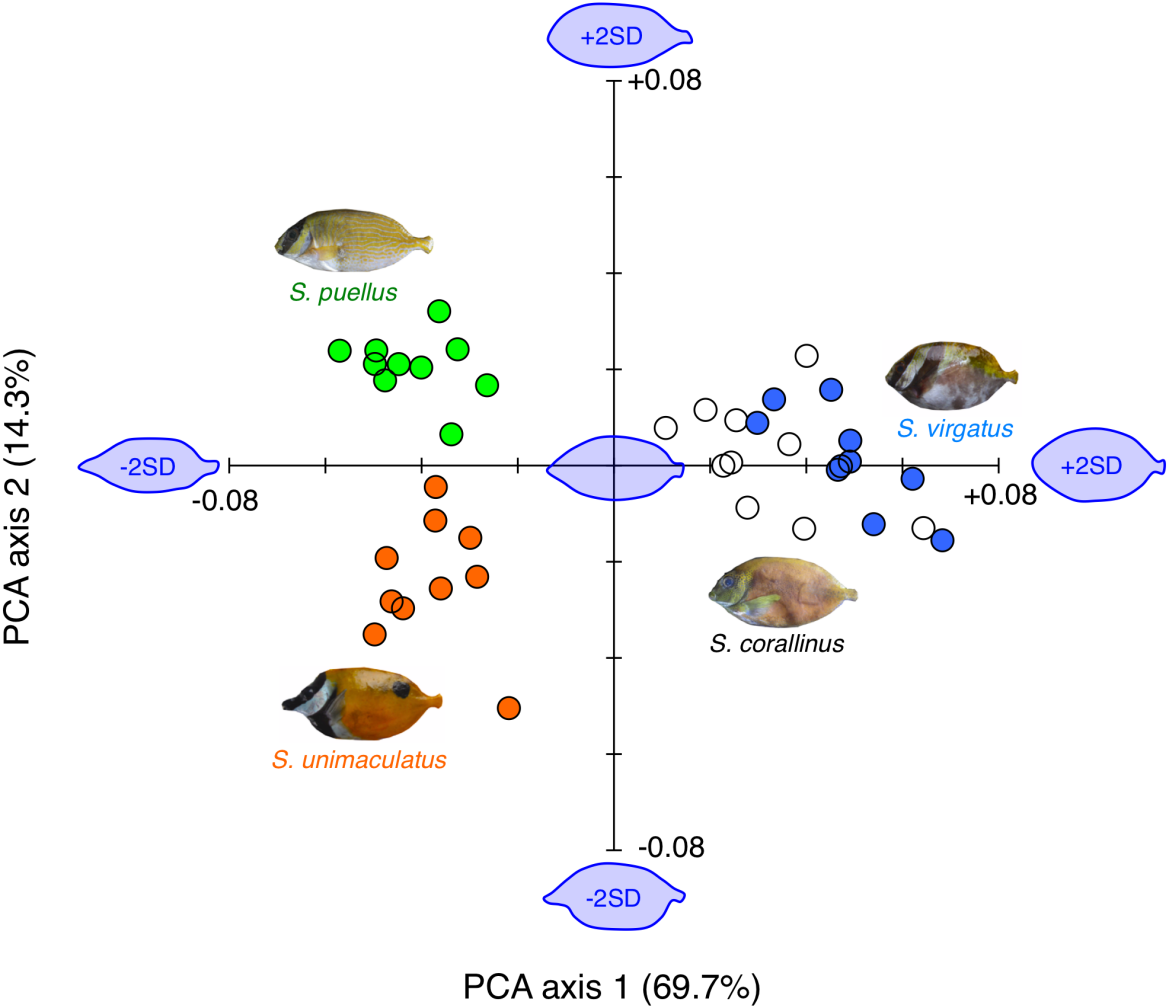
Results of the principal component analysis (PCA) of body shape variations among the four rabbitfish species. The estimated fish body shapes that were constructed using the Fourier coefficients [−2 standard deviations (SD), mean, and +2 SD] are also shown along PCA axes 1 and 2 (blue fish images). Orange circles: *Siganus unimaculatus* (*n* = 10); green circles: *S. puellus* (*n* = 10); white circles: *S. corallinus* (*n* = 10); blue circles: *S. virgatus* (*n* = 10).

Overall, the body shapes of the fishes could be divided into three types: (1) slender body with a protruding snout (*S. unimaculatus*), (2) slender body with a low degree of snout protrusion (*S. puellus*), and (3) relatively deeper body with a low degree of snout protrusion (*S. virgatus* and *S. corallinus*) (Fig. 6).

The two-dimensional plot of the two PCA axes revealed that a positive value along PCA axis 1 indicated a deeper body and a low degree of snout protrusion, while a negative value indicated a shallower body and a protruding snout. These differences explained 69.7% of the observed body shape variation. *Siganus virgatus* had significantly higher scores for PCA axis 1 than the other three species, and *S. corallinus* had significantly higher scores than *S. unimaculatus* and *S. puellus* (*S. virgatus* > *S. corallinus* > *S. unimaculatus* and *S. puellus*; Games-Howell test, *p* < 0.05).

A positive value along PCA axis 2 indicated a low degree of snout protrusion, while a negative value indicated a protruding snout. These differences explained 14.3% of the observed body shape variation. *Siganus puellus* had significantly higher scores for PCA axis 2 than the other three species, whereas *S. unimaculatus* had significantly lower scores than the other three species (*S. puellus* > *S. virgatus* and *S. corallinus*  >  *S. unimaculatus*; Games-Howell test, *p* < 0.05).

### Behavioral interactions

**Figure 7:**
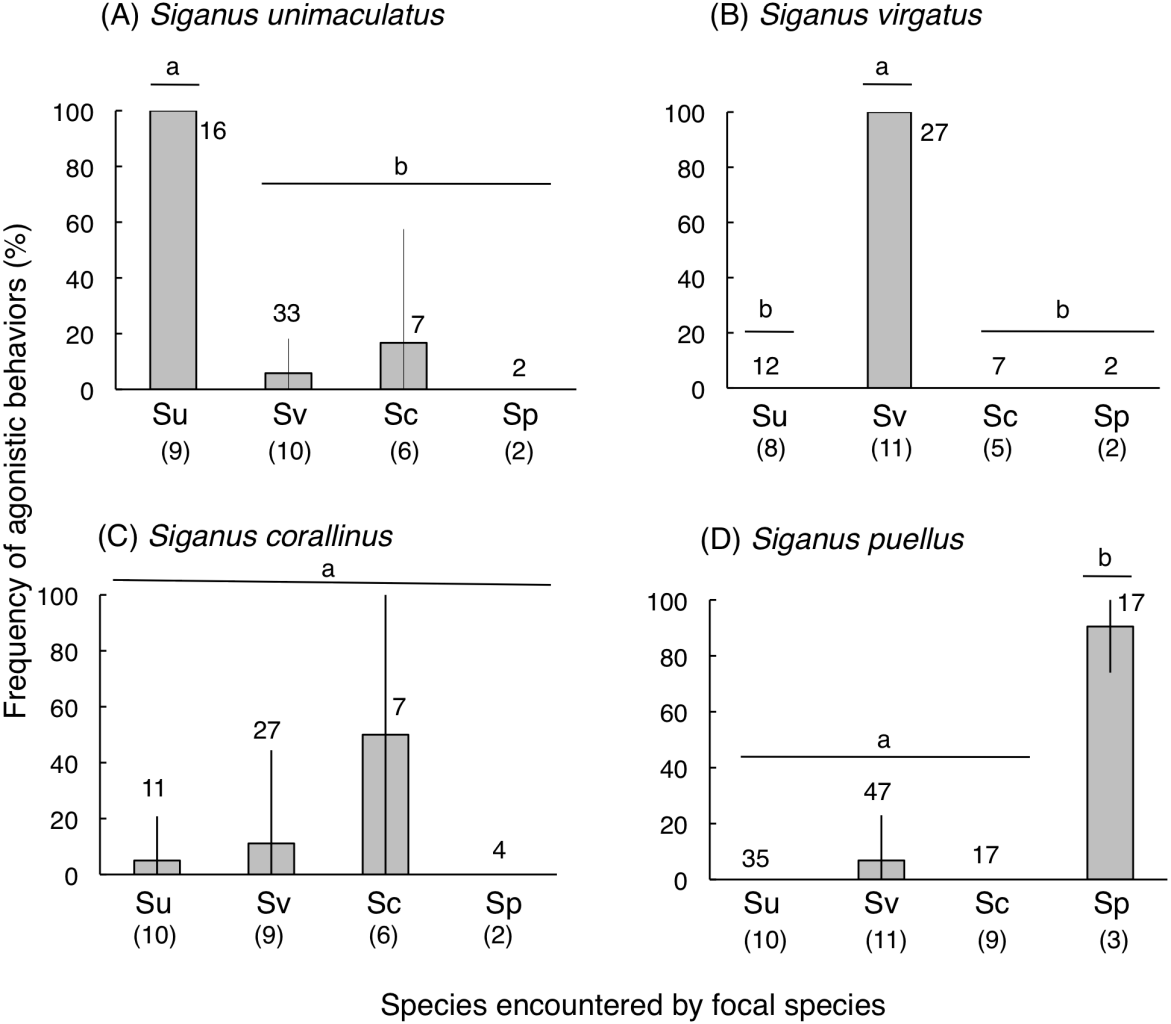
Frequency of “agonistic behaviors” among the four rabbitfish species (see ‘Materials and Methods’ for definition). (A) *Siganus unimaculatus*, (B) *S virgatus*, (C) *S. corallinus*, (D) *S. puellus*. Twelve pairs of fish were observed for each species. Species names are abbreviated as follows: Su, *Siganus unimaculatus*; Sv, *S. virgatus*; Sc, *S. corallinus*; Sp, *S. puellus*. Numbers in parentheses and above the bars represent the number of encountered pairs and the total number of encounters, respectively. Bars that are connected by a line with the same lower-case letter are not significantly different (Scheffé test, *p* > 0.05).Vertical bars represent standard deviations.

*Siganus unimaculatus*, *S. virgatus*, and *S. puellus* showed significantly greater frequencies of agonistic behaviors during conspecific encounters than heterospecific encounters (one-way ANOVA and Scheffé test, *p* < 0.0001 for all three species) (Fig. 7). *Siganus corallinus* also exhibited the same trend, although this was not significant.

## Discussion

### Size-specific spatial distribution in relation to habitat characteristics

Nanami (2015) previously investigated the fine-scale size-specific spatial distribution of the rabbitfish species *S. unimaculatus*, with a focus on the spatial arrangement of its home ranges and the effect of body size on these. However, this study did not consider the relationship between the large-scale spatial distribution and environmental characteristics. Furthermore, although Hoey, Brandl & Bellwood (2013) conducted a detailed analysis of the spatial distributions of 11 rabbitfish species inhabiting the Great Barrier Reef, investigating differences between three shelf regions (inner-shelf, mid-shelf, and outer-shelf) and four habitats (back reef, reef flat, reef crest, and reef slope), they did not consider size-specific spatial variations in relation to substrate characteristics. In the present study, the size-specific spatial distributions of four rabbitfish species were examined over a scale of several to tens of kilometers on an Okinawan coral reef, representing the first such study in this region.

The findings of the present study suggested that inter-specific variation in the spatial distribution was higher for small individuals than for large individuals. It appears that sheltered areas with a greater coverage of branching and bottlebrush *Acropora* spp. may act as nursery grounds for the four rabbitfish species (sensu (Beck et al., 2001). The fine complexity of these coral species would provide a suitable habitat and refuge space for the fishes, likely reducing mortality by predation (Beukers & Jones, 1998; Almany, 2004; Nanami et al., 2013). By contrast, large individuals of all four species were found in rocky areas. Thus, habitat partitioning does not appear to be the main mechanism that allows the coexistence of these species.

### Food item partitioning in relation to feeding behavior

The findings indicated that food item partitioning occurs among the four study species, with *S. unimaculatus*, *S. virgatus*, and *S. puellus* in particular showing clear differences in the food items taken without any evidence of habitat partitioning. Such food item partitioning among species of the same genus has also been found in other families (Nanami & Shimose, 2013; Brandl, William & Bellwood, 2015; Liedke et al., 2017).

It has previously been shown that body shape characteristics can affect the microhabitat that is used for foraging (Brandl, Hoey & Bellwood, 2014; Brandl, William & Bellwood, 2015; Brandl & Bellwood, 2016) and the food items that are taken (Carpenter, 1996; Nanami & Shimose, 2013). The present study demonstrated the species-specific use of feeding substrates among the four rabbitfish species. The findings also suggested that the slender body and protruding snout of *S. unimaculatus* lend themselves to use of a concave substrate, whereas the stouter body shape with low level of snout protrusion in *S. virgatus* meant that it rarely used a concave substrate for feeding. *Siganus corallinus* did not exhibit any substrate-specific use for feeding and took both brown foliose algae and red styloid algae, which was intermediate between *S. unimaculatus* and *S. virgatus*. Since *S. corallinus* has similar (albeit significantly different) body shape characteristics to *S. virgatus*, it may alter its main feeding substrate to reduce inter-specific competition, which may also explain why its main food items were intermediate between *S. unimaculatus* and *S. virgatus.*

Overall, these results suggest that *S. unimaculatus* and *S. virgatus* partition their feeding substrate according to their body shape characteristics, which results in them taking different main food items, enabling their coexistence without any substantial difference in spatial distribution. By contrast, the greater use of flat substrates by *S. corallinus* may enable its coexistence with *S. unimaculatus* and *S. virgatus*. A fine (i.e., species-level) taxonomic classification of food items may provide further detail around the mechanisms that allow these three species to coexist.

*Siganus puellus* mainly fed on sponges, suggesting that it could use all three substrates without experiencing any inter-specific competition. This species has a slender body with a low degree of snout protrusion, which may enable the use of all three substrates. *Siganus puellus* also exhibited a lower feeding rate and average number of foraging bouts than the other species, possibly due to the sponge density being lower than the algal density.

Another aspect that needs to be considered when examining inter-specific differences in food items is the ability to digest and assimilate algae (Clements & Choat, 1995). Therefore, since red and brown algae have been shown to have considerably different carbohydrate compositions (Stiger-Pouvreau, Bourgougnon & Deslandes, 2016), inter-specific differences in the ability to digest the respective food items among the four rabbitfish species should be investigated in the future.

### Frequencies of agonistic behaviors

Nanami (2015) previously revealed that individuals of *S. unimaculatus* have distinct home ranges that are maintained by intraspecific interactions among conspecific pairs. However, few studies have shown the degree of inter- and intraspecific behavioral interactions among multiple rabbitfish species. In the present study, all four species exhibited a greater frequency of conspecific than heterospecific agonistic behavior, suggesting that intraspecific competition for food items and foraging substrates is greater than inter-specific competition for these resources. Overall, such intraspecific agonistic behavior would enhance the syntopic distribution of the four rabbitfish species.

Nanami (2015) showed that similar-sized conspecific pairs of *S. unimaculatus* did not have overlapping territories whereas no such pattern was found for dissimilar-sized conspecific pairs. However, no data on the spatial arrangement of home ranges are available for *S. virgatus*, *S. corallinus*, and *S. puellus*. Therefore, further studies are needed to clarify why conspecific agonistic behavior was frequently observed for all four rabbitfish species.

It should be noted that other herbivorous species that feed on macroalgae and turf algae (e.g., in the families Kyphosidae and Acanthuridae) may also compete with the rabbitfish species for food (Clements & Choat, 1995; Choat, Clements & Robbins, 2002). No encounters with non-rabbitfish herbivores (e.g., *Kyphosus* and *Naso* spp.) that are considered potential competitors were observed during the field observations, suggesting there may be spatial differences between these species. However, to comprehensively understand the mechanisms that allow the four rabbitfish species to coexist, more precise studies are needed on the spatial distributions and feeding ecologies of non-rabbitfish species.

## Conclusion

The purpose of the present study was to reveal the size-specific spatial distributions, feeding ecologies, and behavioral interactions of four rabbitfish species to better understand the underlying mechanisms that enable their coexistence. The findings also fill several empirical gaps in our understanding of rabbitfish ecology. It was found that all four species had size-specific spatial distributions, suggesting that sheltered sites with a greater coverage of branching and bottle-brushed *Acropora* spp. act as nursery habitat. However, no clear habitat partitioning was observed among the four species, suggesting a syntopic spatial distribution. By contrast, there was clear evidence of both food item and feeding substrate partitioning among the four species, which were likely related to body shape characteristics as well as conspecific aggression. Together, these findings suggest that the coexistence of these rabbitfish species is maintained by food item partitioning, which may be enhanced by their behavioral characteristics (i.e., feeding substrate use and conspecific agonistic behaviors).

##  Supplemental Information

10.7717/peerj.6145/supp-1 Table S1 List of benthic invertebrates (except for sponge) that were found as food items of four rebbitfish species Values represent percentage (%) of the food items. For main food items, see Fig. 6.

10.7717/peerj.6145/supp-2 Supplemental Information 1 Fig. 2 raw data

10.7717/peerj.6145/supp-3 Supplemental Information 2 Fig. 3 raw data

10.7717/peerj.6145/supp-4 Supplemental Information 3 Fig. 4 raw data

10.7717/peerj.6145/supp-5 Supplemental Information 4 Fig. 5 raw data

10.7717/peerj.6145/supp-6 Supplemental Information 5 Fig. 6 raw data

10.7717/peerj.6145/supp-7 Supplemental Information 6 Fig. 7 raw data

## Additional Information and Declarations

### Data Availability

The following information was supplied regarding data availability: The raw data are provided in the Supplemental Files.
